# Does a Type of Inciting Antigen Correlate with the Presence of Lung Fibrosis in Patients with Hypersensitivity Pneumonitis?

**DOI:** 10.3390/jcm13175074

**Published:** 2024-08-27

**Authors:** Kamila Deutsch, Katarzyna B. Lewandowska, Agata Kowalik, Iwona Bartoszuk, Piotr Radwan-Röhrenschef, Małgorzata Sobiecka, Małgorzata Dybowska, Witold Z. Tomkowski, Monika Szturmowicz

**Affiliations:** 1st Department of Lung Diseases, National Research Institute of Tuberculosis and Lung Diseases, Płocka 26, 01-138 Warsaw, Poland; kamila.blaszczyk4@gmail.com (K.D.); m.sobiecka@igichp.edu.pl (M.S.); w.tomkowski@igichp.edu.pl (W.Z.T.); monika.szturmowicz@gmail.com (M.S.)

**Keywords:** hypersensitivity pneumonitis, environmental exposure, inciting antigen, precipitins, fibrosis

## Abstract

**Introduction:** Hypersensitivity pneumonitis (HP) is an interstitial inflammatory lung disease that develops as a result of exposition to various, mostly organic antigens. In some patients, fibrotic HP is diagnosed. Factors predisposing to the development of fibrotic lung disease in HP patients are not well documented in the literature. The genetic susceptibility of the patient, type of inciting antigen, and type of exposure, as well as various demographic and clinical variables, may influence the fibrotic process. **Aim:** The aim of the present study was to investigate whether the type of inciting antigen increases the risk of fibrotic lung disease in HP patients. **Methods:** Clinical data of consecutive patients with HP diagnosed between 2019 and 2023 were retrospectively reviewed. The exposition to the inciting antigens was investigated by the standardized questionnaire. Recent HP classification into fibrotic (fHP) and non-fibrotic (non-fHP) types was applied. **Results:** Sixty-six patients diagnosed with HP were analyzed. All patients filled out the exposure questionnaire, and 62 (94%) reported at least one possible exposure. The most prevalent exposures reported were avian, water systems, feather duvets, and hay/straw. Exposure to avian antigens as well as to coal/biomass heating were significantly more prevalent among patients with fHP compared to those with non-fHP (70% vs. 40%, *p* = 0.03 and 27% vs. 5%, *p* = 0.04, respectively). Nevertheless, in the multivariate analysis, older age at diagnosis was the only factor influencing the development of fHP (OR 1.064, 95% CI 1.004 to 1.138, *p* = 0.04). Reported avian antigen exposure correlated well with positive precipitins to avian antigens, whereas no correlation was found between hay/straw exposure and positive antibodies to termophilic actinomycetes. **Conclusions:** Exposure to birds and coal heating was the most frequently present factor in subjects with fHP, but only older age at diagnosis remained a significant fHP predictor in the multifactor analysis.

## 1. Introduction

Hypersensitivity pneumonitis (HP) accounts for 1.5 to 47% of all interstitial lung diseases (ILDs), depending on geographical area and the patients’ ancestry [1]. The recommendations addressing the problem of the recognition of HP were released in 2020 and 2021 to guide respiratory specialists through the labyrinth of diagnostic issues [2,3]. Based on these recommendations, two types of HP, fibrotic (fHP) and non-fibrotic (non-fHP), have been distinguished. Despite this, HP diagnosis, and especially that of its fibrotic type, remains difficult and challenging. Therefore, HP should be included in the differential diagnosis of almost all fibrotic ILDs [2].

Establishing exposure to environmental antigens remains a crucial step in HP evaluation [3]. Exposure is usually assessed during the medical interview with the patient. Several structured questionnaires were proposed to increase the accuracy of interviews, but none were validated and widely implemented [4,5]. Moreover, antigen exposure is also reported in patients with other ILDs [6]. Detection of serum antibodies against particular antigens is another possibility to confirm the exposure [7]. Commercial tests enable the detection of antibodies to only a few common antigens; thus, their sensitivity is not sufficient in some patients, e.g., those with professional exposures [8,9]. More specific tests may be produced using antigens obtained from the patient’s environment. Nevertheless, such in-house tests are usually not validated [10]. Antigen challenge tests may confirm the causative agent in patients with HP, but they are expensive, time-consuming, and may be dangerous to tested patients if the reaction is acute [11,12].

A considerable portion of HP patients develop the fibrotic type of the disease [13]. The prediction of progressive lung fibrosis in HP patients is difficult. Fibrotic HP may mimic idiopathic pulmonary fibrosis (IPF), especially if the evoking antigens are not established [14]. Patients with unknown exposures more often develop lung fibrosis and have worse survival than those in whom the exposure may be confirmed [15]. The data on connections between the type of inciting antigen and HP phenotype are scarce and conflicting.

Thus, the aim of the present retrospective study was to investigate whether the type of inciting antigen, together with demographic and clinical variables, can predict the development of fibrotic lung disease in HP patients.

## 2. Materials and Methods

### 2.1. Study Group

Consecutive HP patients admitted to the 1st Clinic of Lung Diseases of the National Research Institute of Tuberculosis and Lung Diseases in Warsaw, Poland, between 1 March 2019 and 31 December 2023 entered the present study. The diagnosis of HP was made by a multidisciplinary team based on the clinical and radiologic evaluation according to current American Thoracic Society/Japanese Respiratory Society/Asociación Latinoamericana de Tórax (ATS/JRS/ALAT) guidelines [2]. Clinical and demographic data were elicited retrospectively from the institution’s medical database. Exposure to the inciting antigens was investigated by the semi-structured questionnaire published by Vasakova et al. [4], used with the author’s permission. The majority of patients had blood samples tested for the presence of the precipitating antibodies against chicken, duck, turkey, pigeon, and parrot antigens, as well as thermophilic bacteria present in wet hay or straw.

Recent HP classification into fibrotic and non-fibrotic types, according to ATS recommendations, was applied [2].

The potential influence of demographic variables, the number and type of inciting antigens, and bronchoalveolar lavage (BAL) characteristics on the development of fibrotic HP was assessed.

### 2.2. Questionnaire

Patients were asked to provide details of their exposures using the above-mentioned self-administered, semi-structured questionnaire. After analyzing all patients’ responses, the most common antigens reported by the patients were chosen and assigned into groups: poultry, pigeons, other birds, feather duvets, hay/straw, mold, water systems, biomass and/or coal house heating, working on building renovations, and other occupational exposures (wood, textiles).

### 2.3. Precipitins

Serum-precipitating antibodies against specific antigens were tested using immunodiffusion in the agar gel method (Ouchterlony method) [7]. The antigens used included the mix of thermophilic actinomycetes (*Ervinia herbicola*, *Thermoactinomyces vulgaris*, *Thermopolyspora polyspora*) and protein antigens from bird droppings (pigeons, hens, ducks, parrots, turkeys), which are called further avian antigens. The antigens were placed in the wells on agar gel at 1 cm from the central well containing serum tested. After the incubation period of 5 to 7 days at 27 °C, the plates were washed, dried, and stained in 0.1% light green solution. After 24 h, the results were obtained—positive if the precipitation lines representing antigen–antibody complexes were visible or negative if not [7].

### 2.4. High-Resolution CT Scan (HRCT)

In all HP patients, high-resolution computed tomography of the lungs (HRCT) was performed as part of regular assessment. The results were reported as fHP or non-fHP according to the international ATS/JRS/AlAT guidelines [2]. fHP was diagnosed in patients presenting the signs of lung fibrosis in HRCT scans, i.e., reticular opacities, traction bronchiectasis, a decrease in lung volume or honey-combing, accompanying the signs of small airway involvement (centrilobular nodules, ground glass opacities, air trapping or three densities sign) [2].

### 2.5. BAL Fluid Cellular Analysis

BAL fluid evaluation was performed as a part of routine diagnostic assessments according to actual recommendations, and the results were collected from the hospital database. The detailed procedure was described elsewhere [16]. BAL was performed during bronchofiberoscopy according to the ATS guidelines [17]. The fiberoptic bronchoscope was wedged in the subsegmental bronchus, usually in the middle lobe. Then, up to 200 mL of warmed-up 0.9% saline solution was instilled through the working channel of the bronchoscope in equal aliquots and then sucked out with the use of the 20 mL syringe. The recovered fluid was filtered and centrifuged for 15 min at a temperature of 4 °C and at a speed of 400 rotations per minute. The total cell count was assessed in a Bürker chamber. Differential cell count was performed after May–Grunwald–Giemsa staining of the slides in the light microscope [17].

The observation period was censored on 31 December 2023. The disease duration time was calculated from the diagnosis to the date of censoring, lung transplantation, or death.

### 2.6. Statistical Analysis

Statistical analysis was performed using GraphPad Prism version 10.0.1 (170), 25 July 2023 (GraphPad Software, LCC, San Diego, CA, USA). Continuous variables were presented as means ± SD (for normally distributed variables) or medians and interquartile ranges (for variables with distribution other than normal). Categorical variables were presented as numbers and proportions (%). Between-group comparison for continuous variables in two groups was assessed with the T-Student test or Mann–Whitney test, where appropriate. Chi-square test or Fisher’s exact test were used to compare the distribution of categorical variables depending on the group’s quantity. The correlation between serum precipitins and reported exposures was measured using Spearman’s correlation coefficient. *p* values of <0.05 were considered statistically significant. The role of different variables in fibrotic HP prediction was analyzed using multiple logistic regression.

### 2.7. Regulatory Board Approval

This study was approved by the Institutional Ethics Committee of the National Research Institute of Tuberculosis and Lung Diseases (Approval No KB-19/2019, date of approval 27 February 2019). All patients provided written informed consent to participate in this study.

## 3. Results

In total, 66 HP patients, 27 (40.9%) males and 39 females (59.1%), entered this study. Thirty-two (48.5%) of the responders were incident cases (diagnosed in 2019 and later), and thirty-four (51.5%) prevalent cases (diagnosed before 2019). The data on incident cases were evaluated prospectively, whereas, on prevalent cases, they were retrospectively extracted from the hospital database.

The subjects’ median age was 59 (IQR 46.8–67), their median duration of symptoms before the HP diagnosis was 24 months (IQR 11.25–63), and their median follow-up time was 51.5 months (IQR 19.75–80.5).

Forty-six patients were diagnosed with fHP, and twenty with non-fHP. Fifty-five subjects had BAL performed; the median proportion of lymphocytes in BAL was 38.8% (IQR 26.9–52.6).

The baseline characteristics of the study group are presented in Table 1.

**Figure 1:**
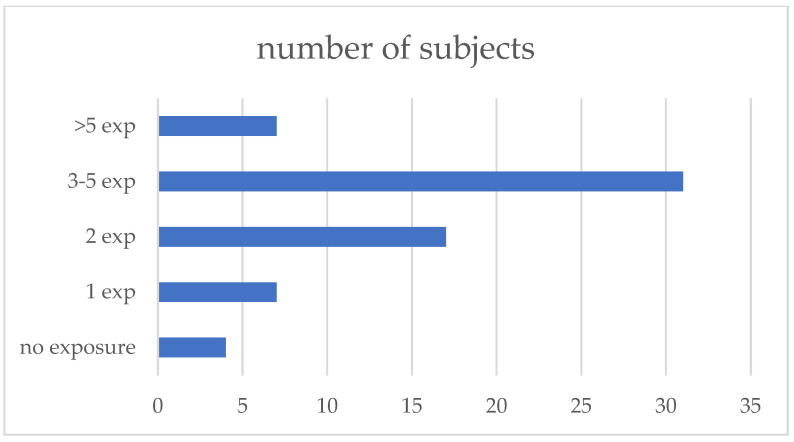
Number of exposures reported.

All patients filled out the exposure questionnaire, and 62 (94%) reported at least one possible exposure; the median number of exposures reported was three (IQR 1–7). The exposures were subsequently grouped into 10 categories: poultry, pigeons, other birds (parrots, canary birds, etc.), feather duvets, hay/straw (farming-related exposures), molds, water systems, biomass or coal house heating, occupational exposures to wood, textiles, and other dust, and working on the renovation of the buildings (Figure 1, Table 2).

In 28 responders (47.5%), precipitating antibodies against avian antigens (chickens, ducks, turkeys, pigeons, and parrots) were present, and in eight (12.9%), antigens related to farmers’ lung (mix of thermophilic actinomycetes) were present.

We compared the characteristics of the 46 patients with fHP and 20 patients with non-fHP (Table 1 and Table 2). There were no differences between those groups regarding sex, smoking status, and the duration of symptoms before the HP diagnosis. The proportion of lymphocytes in BAL fluid in fHP patients was significantly lower than in those with non-fHP (35.85% vs. 51%, *p* = 0.007).

Patients with fHP tended to be older at diagnosis than those with non-fHP (60 vs. 52.5 years of age), but the difference was insignificant (*p* = 0.06). No significant differences were found between fHP and non-fHP patients regarding specific types of exposure. Nevertheless, if the patients with exposures to at least one avian antigen were analyzed together, we found a significant predominance of the participants reporting this exposure in the group of fHP compared to non-fHP (69.6 vs. 40%, respectively, *p* = 0.03). Additionally, there was a trend towards a more frequent exposure to biomass/coal house heating reported by patients with fHP compared to those with non-fHP (27.3 vs. 5%, respectively, *p* = 0.05).

When analyzing any precipitin positivity, there were no differences between patients with fHP and non-fHP. Nevertheless, patients with fHP tended to show precipitin positivity against avian antigens more often than those with non-fHP with borderline significance (56.1 vs. 27.8%, respectively, *p* = 0.05)

Reported exposure to birds’ antigens correlated well with the positive serum-specific G immunoglobulins (ssIgGs) against avian antigens (Spearman’s corr. coeff. 0.342, *p* < 0.05), whereas there was no correlation between the reported farming exposure and the presence of precipitins to thermophilic actinomycetes.

In the multiple logistic regression model, the only factor increasing the odds of developing fHP was older age at diagnosis (*p* = 0.04). The predictive value of BAL lymphocytosis did not reach statistical significance—Table 3.

## 4. Discussion

The fibrotic type of HP develops in some patients exposed to environmental antigens and it is combined with a significant worsening of life expectancy compared to those with non-fHP [18,19,20]. Genetic predisposition, smoking, and other inhaled particle exposures may also play a role in the development of progressive, fibrotic HP [21]. According to recent publications, progressive lung fibrosis concerns 40–58% of fHP patients [22]. Exposure to volatile organic antigens is the most important factor in fHP pathogenesis. Nevertheless, the role of specific organic antigens in fHP development was not confirmed.

Thus, the aim of the present study was to investigate the possible association between fibrotic HP recognition and the type of antigen exposure and cigarette smoking, as well as various demographic factors.

Questionnaires may be helpful in establishing exposures in patients with HP; nevertheless, no validated questionnaires are available worldwide to date [23,24,25]. The data on antigen exposures in our cohort were assessed using a semi-structured self-administered questionnaire published by Vasakova et al. [4] (online data supplement, table E2). The majority of the patients from our group (94%) reported at least one exposure, with a significant proportion reporting exposures to multiple antigens. A similar high proportion of positive responses (96%) was noted by Barnes et al. when using the questionnaire in Australian patients with HP [23].

Most patients in our study group were exposed to water systems. Exposures to certain bacterial and fungal antigens in water aerosols are difficult to confirm and require the cooperation of microbiologists and occupational health specialists [26]. Therefore, a properly constructed questionnaire may be crucial in that setting. Other frequent exposures reported in our study group were feather duvets, farming, and poultry. After combining all reported avian antigens, this was the most prevalent exposure. Avian and mold antigens have also been the dominating causes of HP development in other groups of patients [27,28]. Barnes et al. investigated ILD specialists about the most frequent exposures to build the instrument facilitating patients’ interviewing and finding the causative agents of the disease [5]. Nineteen different antigen sources were indicated as the most important: mold/hay/silage, water systems damage, hot tub, standing water, visible or smelly mold, instruments, moldy wood, air conditioners, birds/feather/droppings, feather products, farming, wood production, food production, isocyanates, metal working fluids, vapors, gases, and fumes [5].

Many authors have delineated the importance of identifying causative antigens in HP patients [15,29,30,31,32]. Once the exposure is identified, the plan of antigen avoidance may be discussed with the patient. It was proven that antigen avoidance resulted in an increase in respiratory parameters and improved survival in patients with both non-fibrotic and fibrotic HP [32,33]. On the other hand, patients with unidentified inciting antigens were diagnosed more frequently with fibrotic HP and had a worse prognosis [27,30,32]. In our group, only four patients reported the absence of possible antigen exposure. Three of them had fHP, but the numbers were too small to draw conclusions.

The published data concerning the significance of antigen type in developing fHP and reduced survival were scarce and conflicting. In our study group, exposure to avian antigens and using biomass/coal for house heating were more prevalent in the fHP group than in the group without fibrosis. These findings are in line with the data published by Hanak et al., who presented an increased prevalence of avian exposure in the HP patients with lung fibrosis [34]. Coal heating is a known risk factor for different chronic lung diseases [35] and using other solid and biomass-based fuels is quite popular in our country. Adams et al. found no relationship between the type of antigen and survival in the group of 155 HP patients, of whom 13% died and 12% had lung transplantations during the median three years of observation [29]. On the other hand, there are some data suggesting a worse prognosis in bird fanciers compared to farmers’ lung patients [36]. The discrepancy between the results of various studies may be partly explained by the different exposure characteristics. It is supposed that chronic exposure to low concentrations of organic antigens may predispose HP patients to lung fibrosis. Our patients self-reported the exposures, and the temporal relationship between exposure and symptoms was not assessed.

Sixty-one percent of patients from our study group who reported bird exposure had positive precipitins against avian antigens. Positive avian precipitins correlated with birds’ antigens exposure, and similarly, they were more prevalent in patients with fHP compared to those with non-fHP. On the contrary, only 18.5% of those exposed to farming had positive serum tests against bacterial antigens. This may be due to a lack of mold antigens in our precipitin panel. Despite many methodological problems, the specific antibody assessment in serum remains a helpful method of exposure assessment [37].

Exposure to avian antigens and coal/biomass was more prevalent in fHP compared to non-fHP patients. Nevertheless, antigen type had no independent influence on fibrosis development in our study group. Most patients reported 3–5 exposures, as shown in Figure 1; therefore, analysis of a specific antigen role in the development of fibrotic lung disease might be imprecise. Due to multifactor exposures reported by the patients, it would be reasonable to ask in the questionnaire whether contact with a specific antigen was combined with any new symptoms of disease. The only independent pro-fibrotic factor diagnosed by us was the older age of the patients. Patients diagnosed at a later age might have a longer history of exposure to possibly occult antigens. Nevertheless, the median time from the first symptoms to diagnosis was comparable in fHP and non-fHP patients.

Older age is a well-established risk factor for another fibrotic lung disease—idiopathic pulmonary fibrosis (IPF). The data show that the prevalence of IPF increases with age, and most patients are diagnosed in their sixties [38]. The role of cells’ senescence is now widely investigated. Senescent cells have a decreased capacity for repairing mechanisms and are more sensitive to DNA injuries and inflammatory processes. It is also related to the telomere length, which decreases with age [39]. Those mechanisms may also play a role in the development of lung fibrosis other than IPF, e.g., HP.

Smoking was not widely prevalent in our group, similarly to other presented cohorts of HP patients [30,40], although some researchers presented a proportion of smokers as high as 50% [15,41]. The data on smoking habit, which was less prevalent in fHP compared to non-fHP patients in our cohort, need further evaluation as these are contrary to other authors’ results [15]. This finding might be biased due to the use of pooled data on current and former smokers without resembling the number of packyears.

A significantly lower proportion of lymphocytes in BALF was found in patients with fHP compared to those with non-fHP, although BAL lymphocytosis in both subgroups was higher than usually reported as a cutoff for HP diagnosis [13,42]. This finding is in line with other authors’ observations [42,43].

Our research had several limitations. First, patients filled out the questionnaire without any assistance from medical staff, which could have influenced the positive response rate. Nevertheless, even such a policy resulted in a great number of reported exposures. On the other hand, questionnaires are usually constructed to receive maximum information with minimal time consumption and without medical staff involvement. Second, we did not assess the symptoms’ development as a result of antigens’ exposure, thus confirming the causative relationship between the particular antigen and disease development. Our study indicates the need to include questions in the questionnaire concerning the symptoms combined with antigen exposure to increase its clinical utility.

## 5. Conclusions

In conclusion, we found that the majority of patients with HP reported some kind of exposure to organic dust. Patients with fibrotic HP were more often exposed to avian antigens and biomass/coal house heating, but these exposures were not confirmed as independent factors predictive of lung fibrosis. The only independent factor increasing the odds of fHP was older age at diagnosis. Further prospective trials are needed to reveal the significance of a particular antigen’s exposure (based on new symptoms of the disease combined with antigens’ exposure) as well as the type of exposure (length, concentration) on the development of lung fibrosis in patients with HP. Questions about those data should be added to the questionnaires to increase precision.

## Figures and Tables

**Table 1:** Baseline characteristics of the study group.

Parameter	Whole Group, N = 66	F-HP, N = 46	Non-fHP, N = 20	*p*
Age at diagnosis, median (IQR)	59 (46.75–67)	60 (47.75–68.25)	52.5 (43.25–63.75)	0.06
Male sex, *n* (%)	27 (40.9)	18 (39.1)	9 (45)	0.77
Ever smoker, *n* (%)	15 (22.7)	8 (17.4)	7 (35)	0.19
**BAL lymph (%), median, (IQR)**	**38.8 (26.9–52.6)**	**35.85 (23.14–48.25)**	**51 (36.6–66.6)**	**0.007**
Time from symptom onset to HP diagnosis (months), median (IQR)	24 (11.25–63.00)	36 (12–75)	24 (6.25–60.00	0.2

IQR—interquartile range; BAL—bronchoalveolar lavage; F-HP—fibrosing HP; Non-fHP—nonfibrosing HP.

**Table 2:** Exposures’ characteristics.

Parameter	Whole Group, N = 66	F-HP, N = 46	Non-fHP, N = 20	*p*
Antigen exposures reported in the questionnaire, *n* (%)	62 (93.9)	43 (93.75)	19 (95.0)	>0.99
Number of antigens, median (IQR)	3 (2–4.25)	3 (2–5)	2 (1.25–4)	0.09
type of antigen				
Poultry, *n* (%)	29 (43.9)	23 (50)	6 (31.6)	0.27
Farming, *n* (%)	29 (43.9)	19 (41.3)	10 (50)	0.51
Pigeons, *n* (%)	20 (30.3)	16 (34.8)	4 (20)	0.23
Feather duvets, *n* (%)	30 (45.5)	23 (50)	7 (35)	0.29
Molds, *n* (%)	24 (36.4)	18 (31.9)	6 (30)	0.58
Water systems, *n* (%)	36 (54.5)	25 (54.35)	11 (55)	>0.99
**Biomass/coal heating, *n* (%)**	**13 (20.3)**	**12 (27.3)**	**1 (5)**	**0.05**
Occupational exposures, *n* (%)	17 (25.75)	12 (26)	5 (25)	>0.99
**Any avian (poultry, pigeons, other birds), *n* (%)**	**40 (60.6)**	**32 (69.6)**	**8 (40)**	**0.03**
Positive precipitins (any), *n* (%)	30 (46.9)	23 (51.1)	7 (36.8)	0.41
**Positive avian precipitins, *n* (%)**	**28 (47.5)**	**23 (56.1)**	**5 (27.8)**	**0.05**
Positive precipitins against thermophilic actinomycetes (farmer’s lung), *n* (%)	8 (12.9)	4 (9.3)	4 (21.1)	0.21

F-HP fibrosing HP, Non-fHP nonfibrosing HP.4.

**Table 3:** Factors predictive of fibrosis development (multiple logistic regression).

Variable	OR	95% CI (Profile Likelihood)	*p* Value
Lymph in BAL (%)	0.9694	0.9336–1.003	0.08
**Age at diagnosis**	**1.064**	**1.004–1.138**	**0.04**
Smoking	0.2712	0.051–1.281	0.11
Coal heating	1.693	0.2054–36.37	0.66
Farming	0.5749	0.1259–2.450	0.46
Avian antigens	1.928	0.4336–9.237	0.39

BAL—bronchoalveolar lavage, CI—confidence interval.

## Data Availability

Data used in this research are available from the authors on request.
